# Providers’ perspectives on inbound medical tourism in Central America and the Caribbean: factors driving and inhibiting sector development and their health equity implications

**DOI:** 10.3402/gha.v9.32760

**Published:** 2016-11-21

**Authors:** Rory Johnston, Valorie A. Crooks, Alejandro Cerón, Ronald Labonté, Jeremy Snyder, Emanuel O. Núñez, Walter G. Flores

**Affiliations:** 1 Department of Geography, Simon Fraser University, Burnaby, Canada; 2 Department of Anthropology, University of Denver, Denver, CO, USA; 3 Faculty of Medicine, University of Ottawa, Ottawa, Canada; 4 Faculty of Health Sciences, Simon Fraser University, Burnaby, Canada; 5 Instituto Nacional de Salud Pública, Cuernavaca, México; 6 Centro de Estudios para la Equidad y Gobernanza en los Sistemas de Salud, Guatemala, Guatemala

**Keywords:** medical tourism, health services research, qualitative methods, Central America, Caribbean

## Abstract

**Background:**

Many governments and health care providers worldwide are enthusiastic to develop medical tourism as a service export. Despite the popularity of this policy uptake, there is relatively little known about the specific local factors prospectively motivating and informing development of this sector.

**Objective:**

To identify common social, economic, and health system factors shaping the development of medical tourism in three Central American and Caribbean countries and their health equity implications.

**Design:**

In-depth, semi-structured interviews were conducted in Mexico, Guatemala, and Barbados with 150 health system stakeholders. Participants were recruited from private and public sectors working in various fields: trade and economic development, health services delivery, training and administration, and civil society. Transcribed interviews were coded using qualitative data management software, and thematic analysis was used to identify cross-cutting issues regarding the drivers and inhibitors of medical tourism development.

**Results:**

Four common drivers of medical tourism development were identified: 1) unused capacity in existing private hospitals, 2) international portability of health insurance, vis-a-vis international hospital accreditation, 3) internationally trained physicians as both marketable assets and industry entrepreneurs, and 4) promotion of medical tourism by public export development corporations. Three common inhibitors for the development of the sector were also identified: 1) the high expense of market entry, 2) poor sector-wide planning, and 3) structural socio-economic issues such as insecurity or relatively high business costs and financial risks.

**Conclusion:**

There are shared factors shaping the development of medical tourism in Central America and the Caribbean that help explain why it is being pursued by many hospitals and governments in the region. Development of the sector is primarily being driven by public investment promotion agencies and the private health sector seeking economic benefits with limited consideration and planning for the health equity concerns medical tourism raises.

## Introduction

The number of patients traveling internationally for medical care is believed to have increased over the past decade (1–9). This growth is attributed to the improved quality of medical care available worldwide and the relative ease of marketing and researching care online. While the actual number of patients traveling for care is unknown due to poor surveillance and inconsistencies in distinguishing the different modalities of international patient flows, this swell in health service exports has significant implications for the resources and operations of health systems globally (4–9). In this article, we focus on ‘medical tourism’, defined as the intentional, private purchase of elective biomedical services outside of a patient’s country of residence. This definition excludes related, but fundamentally dissimilar, health service-seeking practices such as emergency care sought by vacationers, routine care accessed by expatriates living abroad, and cross-border care arranged for patients by their home health care systems that are regularly included in global estimates of ‘medical tourism’ specifically (9).

Many governments and health care providers worldwide are enthusiastic to develop medical tourism as a service export (1–3). Despite the popularity of this policy uptake, there is relatively little known about the specific local factors and discourses informing development of this sector. While there are studies of established medical tourism sectors, particularly from Southeast Asia (2–5), the existing literature has focused on theorizing the potential economic and health system impacts of the practice (6–9). These analyses are predominately retrospective examinations of successful health services exporters; as such they have inherent limitations on the insights they offer into why medical tourism has taken root where it has and the implications for its development elsewhere.

This article draws from the first comparative case study of medical tourism in the Central American and Caribbean (CAC) region using primary qualitative data. This analysis examines common factors driving and inhibiting the contemporary development of medical tourism in Mexico, Guatemala, and Barbados. These countries are all health service exporters, albeit on very different scales and in distinct socio-economic contexts, and have governments and hospitals working to increase the number of visits by medical tourists (10–12). The varying magnitude of existing medical tourism among the three countries, as a function of health system capacity and quality, was purposely sought to explore the factors driving medical tourism across a range of developmental contexts. In Mexico, stakeholders were limited to Tijuana, Monterrey, and Mexico City as the former are known to be centres of health service exports and the latter is the administrative and economic centre of the country. We report here on the findings from interviews with public- and private-sector stakeholders in these countries’ health, tourism, trade, and civil society sectors that identify common factors informing the industry’s development. These factors illustrate why and how medical tourism is being actively promoted in CAC countries and articulates impacts it may have on regional health equity.

## Methods

This analysis contributes to the second stage of a multi-country case study examining the health equity implications of medical tourism in Mexico, Guatemala, and Barbados. The first stage of the study documented retrospective accounts of existing medical tourism activity and sector development in each study site using secondary data. This stage was conducted over a period of 12 months from June 2012 to June 2013 to provide a baseline understanding of medical tourism activity and discussions in each country. The third stage, conducted in the autumn of 2014, consulted Canadian health system stakeholders about the second stage findings to explore their perspectives on the resulting kinds of responsibilities and planning responses by high-income countries implicated in medical tourism *via* foreign investment and international consumption of health services by their citizens. The second stage comprises 150 semi-structured interviews with stakeholders involved in or affected by medical tourism, drawn equally from each country. Participants were sought from four broad domains: 1) health human resources, 2) government ministries and public companies, 3) the private health and tourism sectors, and 4) civil society. The study protocol prospectively set recruitment targets of 15 participants for the first three professional domains and five participants for the last domain, which were met in all three of the study sites. Participants from the domain of health human resources include individuals involved in certification, training, and regulation of nurses and physicians as well as front-line service delivery. Participants from the public sector comprised those involved in analysing and developing public policy for the trade and health sectors, health system administrators and researchers, and representatives from public investment and trade promotion organizations. Participants from the third domain, the private health and tourism sector, include private hospital administrators, tourism operators, and private tourism, health care, and trade consultants. Lastly, civil society participants were drawn from non-governmental organizations with public health mandates and local journalists and academics.

Potential participants were identified from secondary data collected on medical tourism projects (13–15), professional roles in relevant ministries, organizations, or companies, and rolling recruitment from participants. Each potential participant’s domain was classified by the interviewing researcher and confirmed by the site’s lead researcher. All participants remained enrolled in the study until conclusion.

Ethics approval for the study design was granted by the research ethics boards of the lead researchers in each study site. Study invitations were sent to interview participants in advance clearly explaining the study rationale and goals as well as the potential risks and benefits of participation. The invitations also introduced both the international and local researchers, their affiliations, and their respective research ethics boards, with contact information provided for all parties. Participants were informed of their rights to not answer any questions they want and to withdraw their contribution at any point during the study. These rights were provided both in advance with the study invitation and at the time of the interview for those who agreed to participate. Participants’ consent was sought and documented prior to beginning of each interview, with documented verbal consent provided for in cases where participants requested not to provide signed consent due to cultural norms. The anonymity of participants was guaranteed and protected by researchers throughout the research process. All digital interview files related to the project were centrally stored and circulated securely using a private computer server physically located in the lead researcher’s lab.

The interview guide was created in English and translated into Spanish by a professional translator. The guide comprised a general set of opening questions regarding the participant’s background, health system knowledge, and general knowledge of medical tourism, followed by five sets of questions in key domains of medical tourism development and impact: health human resources, trade and investment, government involvement, the public health sector, and the private health sector. Following the opening questions, each participant was asked only questions from their domain of knowledge so as to increase the specificity of their answers, while reducing frustration by avoiding areas of questioning not relevant to their expertise.

An initial round of fieldwork by RJ, VAC, and JS was conducted in Barbados in the summer of 2013 to establish the usefulness of the interview guide. Interviews were then conducted on-site by AC and a research assistant in Guatemala and EON and three research assistants in Mexico between August 2013 and November 2014. All Spanish language interviews were conducted by local researchers in both Guatemala and Mexico. Interviews across all sites lasted from 30 to 60 min. Interviews were digitally recorded and transcribed, with the Spanish interviews translated to English by the same two Guatemalan translators for consistency and sensitivity to cultural ambiguities.

All transcriptions were uploaded to the qualitative data management software NVivo10 (16) for coding. Following a review of five unique transcripts apiece, the lead investigators independently created an initial coding scheme. A meeting was held to consolidate the codes into a single scheme, which was applied to six transcripts (two from each site) to identify redundant and missing codes and produce a final coding scheme following further discussion among the lead investigators. To maintain coding consistency and coherence across the data set, RJ coded all 150 transcripts. The data coded for ‘drivers’ and ‘inhibitors’ of medical tourism development were reviewed by RJ and VAC to identify and compare cross-cutting themes and issues that emerged across the data set and were discussed with the larger team to seek consensus on interpretation. This thematic analysis, as part of our comparative case study, was made possible through identical sampling and interview schedules used across the three countries.

## Results

The interviews highlighted unique factors in Mexico, Guatemala, and Barbados that are informing the development of the medical tourism sectors in each country. The comparative thematic analysis identified four drivers and three inhibitors common to sector development in all three countries. Tables providing illustrative quotes of these factors accompany each section.

### Factors driving sector development

Stakeholders across Barbados, Guatemala, and Mexico understand medical tourism as, first and foremost, an economic issue. The sector is being developed in order to diversify tourism sectors and create economic returns. Very few stakeholders, even within public health sector authorities, framed medical tourism as an issue of health systems operations and planning. Four interrelated and cross-cutting factors driving the development of medical tourism were raised by stakeholders in all three countries: 1) excess capacity in the private health sector, 2) foreign-trained health workers, 3) international hospital accreditation, and 4) medical tourism promotion and development initiatives by publicly owned investment and export promotion companies.

#### Excess private sector capacity

Interview participants in all countries explained how insufficient local demand for private healthcare has stoked interest among private care providers in bringing international patients to fill underutilized hospital beds. While this lack of local demand was attributed to limited private insurance uptake and financial barriers to private care among local patients, the small population of Barbados was identified as an additional contributing factor because of the limits it places on local demand. Select private facilities with advanced infrastructure and staff with foreign training in all three countries were reported to be planning for or marketing to foreign patients (Table 1).

**Table 1:** Illustrative quotes of excess private sector capacity

Guatemala	‘… we really do have an opportunity here due to our good private local health services, they are not saturated’. (Private Sector – 08) ‘Health and tourism is just an expansion of the private sector in the capacity of occupancy they have. Because right now their occupancy doesn’t even reach 70%’. (Private Sector – 19)
Mexico	‘Private hospitals, in this City and the rest of the country, don’t work to the 100%, we work to the 65%, 70%. If we do great in one month, we might be at 85’. (Private Sector – 02) ‘… there are hospitals [that] have an inventory of available hospital rooms that are not being occupied, or areas of opportunity for their doctors or for specialty areas they have, then they say they can explore and participate in a percentage to direct their efforts to [medical tourism]’. (Public Sector – 10)
Barbados	‘I say to you come set up our clinic and yes there are thirty percent of your clients can be Barbadians but … even if the cases are there the persons to pay won’t be there’. (Public Sector – 01) ‘Without medical tourism the kind of facility that we had in mind … simply could not survive on the million tourists that come in a year and the 280,000 people who live on the island, so it really had to have a medical tourism input […they estimated that 40% of the patient volume would come from overseas.]’ (Private Sector – 07)

#### Foreign-trained health workers

Health workers with foreign training, particularly physicians educated in the United States and Europe, were frequently discussed in relation to medical tourism. Firstly, foreign-trained health workers were framed as indicators of the quality of care available locally and understood to be an asset of marketing medical care to patients from high-income settings. Physicians with international training, particularly those who also own their facilities, were raised as key players in advancing private sector interest in marketing care to foreign patients. In addition to the prestige and interpretability of their qualifications for foreign patients, internationally trained physicians’ English language proficiency and motivation to maintain their skill set while practicing in high-end clinical environments were discussed as factors motivating medical tourism development (Table 2).

**Table 2:** Illustrative quotes of foreign-trained health workers

Guatemala	‘Physicians that leave the country and develop outside of the country have very little local practice, community practice. This and the lack of regulation of all the private system has created corporate companies [with] the capacity to provide services similar to the quality offered abroad for very low prices, where even the physician have been trained in other countries’. (Human Health Resources – 07) ‘[Our medical tourism project] has some of the prestigious specialized physicians in the region. They have received courses and have been specialized in Europe and the United States and have the national and international experience of many years. Our services have the same levels as those countries which are highly developed but at much more accessible costs’. (Private Sector – 50)
Mexico	‘We have a … physician with a lot of capacity [who] had the vision to go train in Italy with a physician who managed all those techniques … [for] bariatric surgery to treat obesity. In the United States these procedures were still under approval [and] during this process the physicians that knew him over there [in the USA] began to refer him patients’. (Private Sector – 12) ‘… the doctors that are brilliant and ambitious and want to learn more, want international recognition, they go abroad, they get specialized and then comeback. So what happens? There are patients that will follow them because they know here they will charge less’. (Public Sector – 07)
Barbados	‘… to service an international patient you have to have an international mentality. And a lot of the people that we hired were people who worked abroad … You know they have to have a bigger picture to be able to treat medical tourists’. (Private Sector – 06) ‘… the medical practitioners within the region are highly qualified, a lot of them are North American trained, but board certified within the region. So it’s a matter now of just seeing where the gaps are … and how we can get to that critical level where we can say yes, this is a sector we can confidently market to whatever target market we want’. (Public Sector – 05)

#### International hospital accreditation

Medical tourism is widely understood to be a ‘pay-to-play’ arena, where international hospital accreditation is the entry fee. Many large private hospitals in Mexico have been internationally accredited, two in Guatemala were seeking accreditation at the time of data collection, a clinic in Barbados has obtained such accreditation and development plans for a new private facility there note that international accreditation will be sought. It was reported that while international accreditation serves as a quality indicator for marketing to international patients, it was also widely understood to permit access to the American health insurance market, without which it would be difficult to be considered a legitimate care provider. Perhaps most significantly, Mexican stakeholders reported that domestic hospital accreditation is currently being harmonized with international hospital accreditation standards to improve care quality and facilitate insurance portability (Table 3).

**Table 3:** Illustrative quotes of international hospital accreditation

Guatemala	‘I used to say to myself, why do need to be accredited if I have a pretty clinic? But there are certain regulations that we must comply with so … we can go with a company or a big insurance company and tell them, we want to sell you this service’. (Private Sector – 47) ‘We are right now fixing with some companies … due to the Obama law [Affordable Care Act], where they give many companies from the United States the power to choose the health services from other countries that are accredited, if they don’t like the prices of the health services in the United States. This is why it is important that we are organized and that we have our accreditations and that we have the support from the government’. (Private Sector – 48)
Mexico	‘Most of the times [accreditations] are necessary because insurance companies are the ones who enforce this. And if hospitals don’t have accreditations, patients aren’t sent to those hospitals’. (Private Sector – 13) ‘… because of the constant requirements done by insurance companies, medical tourism promoters, hospitals, and clinics have tended to obtain certifications with international organizations … it does give more confidence for the patient that comes from abroad’. (Private Sector – 15)
Barbados	‘… the challenge that remains with us is that if a person is going to be traveling from the United States or Canada or any first world country the insurers are saying that the facility where you’re going to must be up to standards’. (Public Sector – 01) ‘… there’s a lot of insurance companies now that are looking at international portability of benefits, so it’s not as exotic as it once was. The thing is to show that you’ve got a quality product and show what your outcomes procedures are, and your accreditation’. (Private Sector – 11)

#### Public promotion of private medical tourism projects

Public trade development corporations that promote exports and foreign investment have all been active in developing public policy and analyses for the sector in all three countries, disseminating information locally and advancing hospital and marketing projects internationally. ProMexico has identified ‘clusters’ of medical facilities in each state that they plan to advertise abroad as the best quality of care available in the country. INGUAT is coordinating private and public sector efforts to export health services from Guatemala while promoting its hospitals internationally. Invest Barbados has organized and attended medical tourism trade shows to promote the sector and facilitated the planning for a new private hospital that will be oriented to foreign patients. These export and investment development corporations are the common lead actors in sustaining the conversation around medical tourism in their home countries and promoting its development (Table 4).

**Table 4:** Illustrative quotes of public promotion of private medical tourism projects

Guatemala	‘Each chamber is going to be in charge of a specific part of the market; but INGUAT is the one that coordinates all the effort that’s being made as a country’. (Private Sector – 13) ‘[INGUAT] are about bringing tour-operators or insurers, people specialized in this kind of tourism, so that they get familiar with Guatemala’s situation about medical tourism. Another [strategy] is being part of different associations, like Medical Travel Association, the one responsible of gathering all specialists in medical tourism’. (Public Sector – 23 and 24)
Mexico	‘… [Promexico] took the leadership, [it does] commercial missions. At the end of this year and on September [it is] about to begin [its] commercial mission to United States with national hospital, large chain to contribute with, it is going to be like a matchmaking in a reunion with American insurance companies to start attack this market’. (Public Sector – 08) ‘[Promexico] had as one the most important activities … a diagnosis of medical tourism in Mexico … a complete report that exposed the prospective, which place did we have on an international level, what were the most important federative entities, what were the hospitals that were participating in this movement, also to know the human resource that was available, the infrastructure, and so on’. (Public Sector – 07)
Barbados	‘… in our promotional literature [Invest Barbados] we included [medical tourism] as one of the new sectors that we’re developing. In our overseas promotions when we speak to groups we’re actively promoting this as one of the areas that we would want to encourage’. (Public Sector – 06) ‘… in 2005/2006 the Caribbean Export Development Agency coordinated a study into services that could benefit the country, benefit Barbados and one of the services recognized or identified was medical tourism. So Invest Barbados took that study onboard and started to seek out the service providers to show them the way, as it were, rather than service providers seeking us out. We [sought] them out because that’s our role’. (Public Sector – 07)

### Inhibitors of health services export

A common, cross-contextual narrative about the challenge of sustaining interest in medical tourism emerged from the Mexican, Guatemalan, and Barbadian stakeholders. Interviewees described an initial period of enthusiasm among private- and public-sector stakeholders for sector development, spurring workshops and inter-sectoral planning. However, in spite of the working groups, policy initiatives, and hospital projects that the enthusiasm for becoming medical destinations spurred, stakeholders consistently described significant setbacks in expanding their medical tourism sectors. Three cross-cutting inhibitors were found to be shared across the sites: 1) the high expense of entering the medical tourism market, 2) incoherent planning within and between the private and public sectors, and 3) structural socio-economic issues such as insecurity and relatively high business costs and financial risks.

#### High expense of market entry

Participants identified the high cost of entry to the international health service market as a large and immediate barrier to sector development. Medical facilities looking to attract international patients were generally understood as having to be aesthetically modern with up-to-date technology, infrastructure, and safety protocols. Costs resulting from international hospital accreditation, both the process itself and upgrading to its requirements, were regularly raised by participants from the private health sector as the most significant barrier. As a result of the perceived high costs of competing for medical tourists, promotion of the sector is largely limited to the best-financed and equipped private facilities (Table 5).

**Table 5:** Illustrative quotes of the high expense of market entry

Guatemala	‘They were telling me that getting accredited is very expensive and it takes a very long time. And both statements are true. The expensive thing is not to pay the license, I pay $1,525 a year for my license. But for me to make the changes to get it I spent another $25,000’. (Private Sector – 47) ‘Accreditations are somewhat expensive. A hospital in Guatemala is about to get certified, but it has taken years and a lot of investment. That’s definitely a barrier … Certifications and advertising are the most terrible barriers we have. If I want my clinic to be able to receive foreigner patients, I have to comply with a lot of requisites’. (Private Sector – 13)
Mexico	‘The Health Board was in contact with the [Joint Commission International] to negotiate and to homologate their certification, avoiding that way that the private hospitals had to realize a double effort, or that [Mexican] certification [would be seen] as second-[best]. One of the problems for [JCI] certification is the high cost that only a few hospitals of the country [could afford]’. (Human Health Resources – 04) ‘Certification … is something in which we cannot be flexible because this is what guarantees certain quality. There are the private hospitals that can [certify] because [they can] invest time … and money. There are hospitals that even had to invest around 2 million pesos or even 5 million pesos in order to do all the necessary adjustments that the certification was asking’. (Public Sector – 07)
Barbados	‘… the availability to funds is not there for everyone. So for a group of local doctors to get together and try to build a hundred million dollar facility it’s not going to happen … Or a twenty million dollar facility, it’s not going to happen’. (Human Health Resources – 08) ‘… [medical tourism] will need infrastructure, it will need training, it will need equipment and all those things require funds and with the economic condition in Barbados now they aren’t available’. (Public Sector – 01)

#### Incoherent sector-wide planning

In each country, participants identified two sources of incoherence in advancing the medical tourism sector. Firstly, intra-governmental responsibilities and goals for the sector are not well defined, with initiatives poorly coordinated between different levels of government, ministries, and public trade promotion corporations. Concerted efforts to bolster medical tourism ultimately failed to outlive the tenure of cabinet ministers who initiated them. Secondly, hospitals and physicians in the private health sector are described as wary of one another’s efforts. In Guatemala and Mexico, existing private hospitals have established rivalries and do not want to benefit competitors through sector-wide coordination. In Barbados, many physicians are wary of large-scale medical tourism projects that plan to import foreign physicians, introducing additional private-sector competition for local patients (Table 6).

**Table 6:** Illustrative quotes of incoherent sector-wide planning

Guatemala	‘… the problem is that there’s no follow up. Once we’ve reached an agreement with a director or head office, or whatever, sometime later he/she gets removed from his/her position and we lose all the progress. It’s like what we had built so far, crumbles. The problem is that there’s no continuity in programs, there’s not enough support’. (Private Sector – 04) ‘… for now there are four organizations at a national level that I know of working in the field of medical tourism and all these are being grouped in a committee by INGUAT … It is still in process of maturity as everyone has their own ideas and there is still no integration of all four organizations to move to specific goals. To be able to change policies and get this going to make it attractive for business, we have to work as a group’. (Private Sector – 08)
Mexico	‘It is important that Tourism, Health, Economics and Promexico organize between them to see what they have to offer and … that we all understand the business theme and the need to organize with business for this to work out. You cannot dictate guidelines from the Government without taking the businessmen into account. … A year ago there was a forum in Tijuana for medical tourism organized by the Government and you could see that there where all of the Secretariats and the bunch of States but where were the businessmen?’ *(*Private Sector – 06) ‘Hospitals are not willing to agree to everything, especially costs. On the other hand, there are hospitals A, B, C, D, to avoid naming. And so A comes and says “I’ll wait what B and C say, to see if I’m willing to agree.” So as long as there is no group work, it’s too difficult to sell [medical tourism … There is no a communion between us’. (Public Sector – 02)
Barbados	‘[Medical tourism promotion] happens but it has not really been as structured as perhaps it could be. And then the other thing too … is that you have silos, so one entity is doing something, the next entity is doing something and they’re not really coming together’. (Public Sector – 3 and 4) ‘… even when the government doesn’t change the Ministers of Health could change … So then the new Minister of Health, he now starts dealing with the whole thing over again … that’s one of the hindrances to [medical tourism] moving forward. The other thing too is … there’s a caution about not having a good feel of how the local physicians would interact [with foreign physicians practicing locally.]’ (Private Sector – 09)

#### Local insecurity and high cost of care

Two perceived socio-economic inhibitors identified by participants were country specific. Insecurity was the most frequent social issue raised by Mexican and Guatemalan participants as inhibiting the development of medical tourism in their countries. It is thought that ongoing local violence would deter international patients. Barbados was distinct from the other sites as low rates of violent crime have been identified as a strength in developing medical tourism. However, the high operating and labour costs relative to Barbados’ regional CAC competitors played a similar (albeit much less prominent) role in participants’ discussions of endemic barriers to sector development (Table 7).

**Table 7:** Illustrative quotes of local insecurity and high cost of care

Guatemala	‘We shouldn’t waste our time in going to the US to promote and sell our packages – even if American Airlines gives us free tickets – because we are more expensive than the rest of the region and no one will want to come. There’s also another matter … it’s very unsafe in the country’. (Private Sector – 16) ‘Our first problem is insecurity … Who is going to come to Guatemala if here you get mugged or killed over a cell phone? So, for example, with a group of brokers we had, we drove them around in a shuttle bus, and we had a police car following us. So what do you think they’re going to think about us if we had a police car behind us?’ (Private Sector – 04)
Mexico	‘One of the things that affected us was the time of insecurity that we lived almost 3 years ago. We saw how the [medical tourism] demand decreased … We still had patients but we had to reinforce that we were located is a very secure area and that nothing had ever happened here’. (Private Sector – 11) ‘We (doctors) can do our job wonderfully, but if you ask about medical tourism in Tijuana, many would rather run away to India before stepping into Tijuana because of the general insecurity situation, in all border [areas]. That’s something the federal, state and all governments in general have to work on together, to bring the country’s security back’. (Private Sector – 14)
Barbados	‘I don’t foresee [medical tourism] happening here … We don’t have the volume because even if it’s cheaper to go, our prices are quite high too, because we’re in close approximation to the States and everything is imported from the States’. (Human Health Resources – 12) ‘… the fact there’s no legislation allowing anybody whose building a medical facility to set it up with those kind of concessions, you bear the brunt of what the local costs are and the local costs are high, very high’. (Private Sector – 15)

## Discussion

Our findings are consistent with key elements of existing accounts of medical tourism development elsewhere (2–4, 17, 18), supporting their relevance and attendant implications in new contexts. In particular, three of the driving factors identified by our analysis, private-sector oversupply, local availability of internationally trained physicians, and international hospital accreditation, echo what has been documented in the literature (2–9, 19, 20). However, participants’ accounts emphasize different facets of each driver that have not been identified in previous analyses, such as the coordination of local hospital standards with international accreditation, the role of international hospital accreditation in supporting internationally portable health insurance, and interest in the sector being driven in part by internationally trained specialists seeking to maintain their skills and practice in resource-intensive clinical settings.

In the CAC region, medical tourism is understood to be a solution to address unused capacity in the private health sector (21, 22). For Guatemala and Mexico, this capacity already exists and is seeking utilization; for Barbados, the demonstrated viability of tapping into the international market to reach a sufficient catchment of private patients is facilitating planning for new private facilities. On the surface, this supports the idea that medical tourism can be a relatively benign source of additional revenue for health systems that bolsters health infrastructure and economic development (8). However, as it is the existence of severely inequitable distribution of medical resources domestically that has given rise to the rationale for private medical providers to turn to international patients, the benefits associated with medical tourism are unlikely to reach beyond the silo of the private health sector without accompanying redistributive policies. Developing and enforcing redistributive policies thus remain a priority for governments pursuing medical tourism if they intend to ensure that their health systems for the local population are strengthened by sector development (3, 23).

The driving role of attracting and retaining highly trained health workers has been highlighted as a key factor in accounts of the development of medical tourism elsewhere (8, 19, 20, 24). Previously, these accounts have focused on internationally trained health workers as internationally marketable assets, framing their role in driving development of the industry as relatively passive resources to be drawn on (1, 18, 24). Our interviewees emphasized the active, entrepreneurial role of internationally trained physicians in stoking interest in the sector. This entrepreneurship was articulated in terms of profit seeking, but perhaps of more interest, also in terms of a desire to operate in excellent clinical environments while retaining and further developing their skills. These findings support the notion that medical tourism is one means to attract and retain health workers who might otherwise emigrate, but tease out the multi-faceted rationale by which it may do so beyond its potential to support higher wages. The findings also raise further questions of how health system planners are to engage the skills of foreign-trained health workers within the local epidemiologic and health system context if medical tourism supports highly skilled practitioners to opt out of treating the most pressing domestic needs (2, 5–7).

The third driving factor identified by the findings, international hospital accreditation, is unsurprising as it is found throughout the existing literature as a standard ‘ingredient’ in the developmental formula for medical tourism (1–8, 25). However, participants clarified the role international hospital accreditation is playing in planning for the sector in the CAC region. While existing discussion of international hospital accreditation focuses on its role as a marketable credential and means to improve and standardize quality of care, many participants emphasized international hospital accreditation as a *precursor* to accessing private insurance funds outside of their countries. International hospital accreditation should thus be understood not only as an emerging standard of care, but also as a precursor to scaling up flows of international patients into the future (21, 26).

The fourth driving factor that emerged from this analysis, promotion and planning for medical tourism by public export development and investment corporations, is not well reflected in existing accounts of sector development. While instances of medical tourism being coordinated and promoted by government agencies are found in the literature (2–5, 21), the parallel roles of the public export and investments promotions corporations in all three countries are striking in their formulaic recurrence and its consequent implications for health policy in each country and across the region. As the most visible government organizations driving medical tourism in CAC are both removed from health systems planning and approaching medical tourism solely as an economic development project, the risk of the sector developing with only cursory consideration of the many health equity concerns medical tourism raises (2–8, 27) is significantly heightened. If well managed by a wide range of organizations and stakeholders, it is argued elsewhere that medical tourism has the capacity to benefit the overall health system in the form of cross-subsidization of care, advanced infrastructure, and support for a wide range of specialities (8, 27). However, the narrow frame of reference and expertise that is informing the development of the sector in CAC reduces the likelihood of achieving these wider systemic gains.

Lastly, the inhibitors raised by participants, those of high costs to market entry, incoherent planning, and endemic socio-economic burdens, raise larger questions of the suitability of a wholesale health services export sector for the CAC region. High costs of market entry will likely restrict the revenues of medical tourism to the already best resourced facilities and further narrow the potential benefits of the sector. Incoherent planning that has proceeded in spurts in all three countries is preventing a comprehensive vision for what the sector aims to achieve, in terms of both economic and health development, its potential pitfalls, and which parties should oversee its development. Neglecting to address these barriers while the underlying driving factors push development of medical tourism ahead will further entrench the inequitable tiers of care that have given rise to the phenomenon.

## Conclusions

There are a number of shared factors shaping the development of medical tourism in CAC that help explain why it is being pursued by many hospitals and governments in the region. Our analysis of 150 stakeholder interviews from Barbados, Guatemala, and Mexico identifies four common driving factors: 1) excess capacity in the private health sector, 2) foreign-trained health workers, 3) international hospital accreditation, and 4) medical tourism promotion and development initiatives by publicly owned investment and export promotion companies. The analysis also identified three common inhibiting factors: 1) the high expense of entering the medical tourism market, 2) incoherent planning within and between the private and public sectors, and 3) structural socio-economic issues.

This study indicates that medical tourism development is primarily being driven by public investment promotion agencies and the private health sector as they seek economic benefits with limited consideration and planning for the health equity concerns medical tourism raises. The factors identified here as currently shaping the industry in CAC should be incorporated into planning for the sector to ensure that the wider potential gains of medical tourism are channeled into the health system as a whole.

## Paper context

The ongoing development of medical tourism has significant global health implications, particularly in regard to health system capacity and the equitable distribution of health resources. This analysis of 150 stakeholder interviews from three Central American and Caribbean countries identifies a confluence of common factors that are prospectively influencing regional interest in medical tourism. Identifying these factors assists in understanding how and why medical tourism is being so widely pursued and can support the creation of policies that enhance equitable outcomes while minimizing the potential harms of the sector.
